# Advances in Lead-Free Piezoelectric Materials for Sensors and Actuators

**DOI:** 10.3390/s100301935

**Published:** 2010-03-10

**Authors:** Elena Aksel, Jacob L. Jones

**Affiliations:** Department of Materials Science and Engineering, University of Florida, 100 Rhines Hall, Gainesville, FL 32611, USA; E-Mail: eaksel@ufl.edu

**Keywords:** ceramics, piezoelectrics, ferroelectrics, electromechanical

## Abstract

Piezoelectrics have widespread use in today’s sensor and actuator technologies. However, most commercially available piezoelectric materials, e.g., Pb [Zr_x_Ti_1−x_] O_3_ (PZT), are comprised of more than 60 weight percent lead (Pb). Due to its harmful effects, there is a strong impetus to identify new lead-free replacement materials with comparable properties to those of PZT. This review highlights recent developments in several lead-free piezoelectric materials including BaTiO_3_, Na_0.5_Bi_0.5_TiO_3_, K_0.5_Bi_0.5_TiO_3_, Na_0.5_K_0.5_NbO_3_, and their solid solutions. The factors that contribute to strong piezoelectric behavior are described and a summary of the properties for the various systems is provided.

## 1. Introduction

Piezoelectric materials are commonly used in sensor and actuator technologies due to their unique ability to couple electrical and mechanical displacements, *i.e.,* to change electrical polarization in response to an applied mechanical stress or mechanically strain in response to an applied electric field [1]. Compared to other electromechanical transduction technologies, piezoelectric materials offer a high pressure per density ratio for actuator devices, high environmental and chemical stability, and capabilities of operating at high temperatures and frequencies. Applications of piezoelectric materials range from buzzers to diesel engine fuel injectors, sonar, ultrasound, and nanopositioners in scanning microscopes.

The most widely used piezoelectric ceramic is lead zirconate titanate (Pb [Zr_x_Ti_1−x_] O_3_), or PZT. One critical disadvantage of PZT is that it contains more than 60 percent lead (Pb) by weight. This large lead content creates hazards during processing (lead volatilizes and is released into the atmosphere), limits applications (e.g., *in vitro*), and is potentially environmentally toxic during disposal. Over the past few years, regulatory agencies world-wide began putting strict restrictions on the use of lead, with the exception of the electronics industry due to the lack of a suitable replacement to PZT [23].

Suitable lead-free piezoelectric materials are still being developed as no single composition has been proposed with properties that are comparable to that of PZT. This review highlights some of the current advances in the development of lead-free piezoelectric materials with a focus on those exhibiting the perovskite structure. The remaining sections of the manuscript are broken down as follows. Sections 2 and 3 contain background information: the fundamentals of piezoelectricity are briefly reviewed in Section 2 and the importance of morphotropic phase boundaries is described in Section 3. Section 4 discusses developments in several different material systems that are based on one particular composition, e.g., BaTiO_3_, Na_0.5_Bi_0.5_TiO_3_, *etc.* Section 5 introduces research advances in solid solutions containing two of these compositions. Section 6 discusses the ternary solid solutions that exhibit useful properties. Many of the material compositions and properties that are discussed in each of the respective sections are also compiled in Table A.1 of Appendix A. A brief summary of the paper is provided in Section 7.

## 2. Fundamentals of Piezoelectricity

The only crystallographic requirement for a material to exhibit piezoelectricity is that it must be non-centrosymmetric. Examples of non-centrosymmetric structures that are used in piezoelectric devices include aluminum nitride (AlN) and quartz. In many piezoelectric materials, a spontaneous polarization also exists due to the separation of negative and positive charge centers in the crystallographic unit cell. A characteristic example of a structure which exhibits a spontaneous polarization is the perovskite structure, ABO_3_. As the perovskite structure is cooled from the high temperature cubic phase (centrosymmetric), it can undergo several different phase transitions and eventually transform to tetragonal, rhombohedral, orthorhombic, or monoclinic structures at various phase transitions. The center atom (B) and the oxygen octahedron (O_3_) displace non-uniformly relative to the corner atom (A), resulting in a non-centrosymmetric structure. The temperature at which the structure transforms from the high-temperature cubic phase to the first structure exhibiting a spontaneous polarization coincides with the Curie temperature. When cooling a material through the Curie temperature, different regions of the material take on different crystallographic orientations of the lower symmetry crystal structure. These different regions are referred to as domains and the regions that separate different domains are referred to as domain walls.

Ferroelectricity is the ability of a material to change its direction of spontaneous polarization in response to application of an electric field. The electric field required for this reorientation to occur is known as the coercive field and typically involves the motion of a ferroelectric domain wall. There is often a distribution of local coercive fields in a polycrystalline material since there may be compositional variations across a grain or different stress states of different grains. The macroscopically observed coercive field (*E_c_*) is the electric field required to obtain zero macroscopic polarization due to compensating positive and negative local polarization states.

Upon cooling from the high processing temperatures required for ceramic materials, polycrystalline ceramics exhibit neither a net macroscopic spontaneous polarization nor piezoelectricity at the macroscopic length scale. This is because the structure is constituted of an equal number of all possible domain orientations, each with a spontaneous polarization oriented in different direction. Since the material contains many domains all oriented in different directions, the local areas of spontaneous polarization cancel each other and the material does not exhibit a net macroscopic polarization. For a polycrystalline material to exhibit piezoelectricity at the macroscopic length scale it must go through a poling process wherein an electric field is applied and the domains are aligned more closely parallel to the electric field direction. After poling, the material will have a net macroscopic polarization parallel to the direction of the poling field and will exhibit piezoelectricity at the macroscopic length scale.

The converse piezoelectric effect describes the strain generated in a piezoelectric material in response to an applied electric field. This effect is written as:

(1) $$S_{i}=d_{\mathrm{ij}}E_{j}$$

where *S_i_* is the electric field induced strain, *E_j_* is the applied electric field and *d_ij_* is the piezoelectric coefficient. The piezoelectric coefficient is truly a third rank tensor, although is written in Equation (1) in reduced matrix notation by representing the mechanical strain as a 1-dimensional matrix with elements *i* = 1, 2…6. The converse piezoelectric effect is exploited in actuator devices.

For sensing applications, the direct piezoelectric effect describes a change in polarization due to an applied stress and is written as:

(2) $$D_{i}=d_{\mathrm{ij}}\sigma_{j}$$

where *D_i_* is the dielectric displacement and *σ_j_* is the applied stress.

In Equation (1) and (2), the coordinate axes are defined by the polarization of the sample and is assigned to the 3-direction. When an electric field is applied parallel to the 3-direction and strain is also measured in the 3-direction, the piezoelectric coefficient of relevance is the longitudinal piezoelectric coefficient, *d_33_*:

(3) $$S_{3}=d_{33}E_{3}$$

The piezoelectric coefficients described by the direct and converse piezoelectric effects are mathematically equivalent. Therefore, the longitudinal piezoelectric coefficient described by the converse piezoelectric effect (Equation (3)) is equivalent to the longitudinal piezoelectric coefficient described by the direct piezoelectric effect:

(4) $$D_{3}=d_{33}\sigma_{3}$$

Another important property of a piezoelectric material is permittivity, which is related to how much electrical potential energy can be stored in a given volume of the material under the influence of an electric field. This is often maximized near phase transitions and its distribution with temperature is broad in relaxor ferroelectric materials. Often, the permittivity is reported relative to the permittivity of free space (*ε_33_^T^/ε_0_*), also called the relative permittivity (*ε_r_*) or often the dielectric constant. Finally, the electromechanical coupling factor (*k_p_*) relates the electrical energy output to the total mechanical energy input or vice versa [4].

The properties of a piezoelectric material can be improved with small addition of substitutional impurities, or dopants, as previously exploited in designing the properties of PZT. Depending on the type of dopant and its position in the unit cell, the structure and properties of a material will change in different ways. In the case of PZT, for example, donor doping involves dopant ions which are more positive in valance than the ions they are replacing and this leads to a “soft” ferroelectric material behavior [5]. Some of the property changes associated with this type of doping are a decrease in the coercive field, increased dielectric constant, and an increased electromechanical coupling factor [5]. On the other hand, acceptor doping in PZT involves a dopant ion which is less positive in valance than the host ion and this leads to a “hard” ferroelectric material behavior [5]. Some of the characteristic changes attributed to this type of doping are a moderately lowered electrical resistivity, higher coercive field, and a relatively lower dielectric constant [5]. “Hard” PZT materials are also often more difficult to pole and depole.

## 3. The Morphotropic Phase Boundary

**Figure 1.**
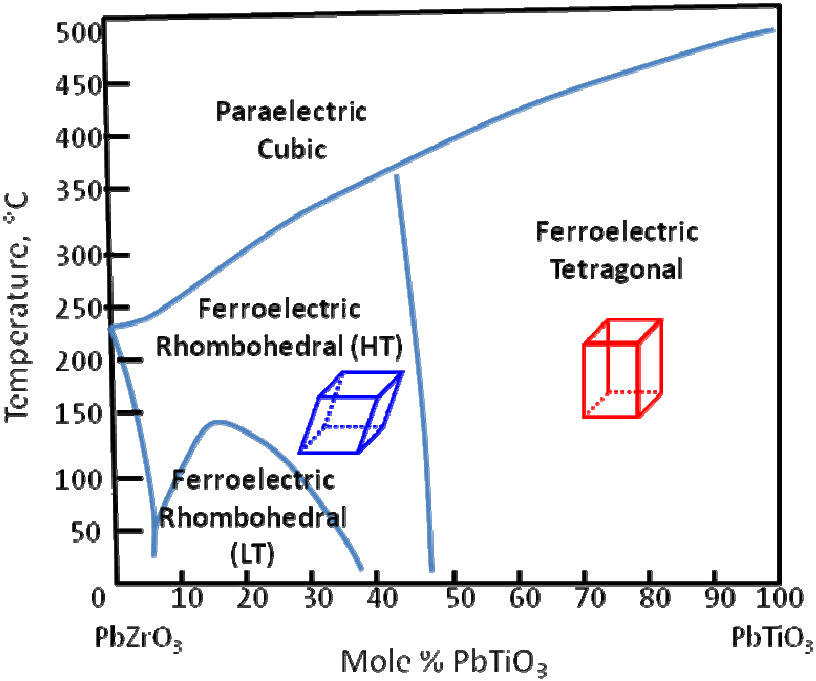
Morphotropic phase boundary in PZT, reproduced from Jaffe *et al.* [8].

The extremely high piezoelectric response of PZT is partially attributed to the presence of a morphotropic phase boundary (MPB). The MPB describes the boundary that separates regions of different symmetries and can be crossed through a change in composition [6]. In 1954, Jaffe *et al.* found enhanced piezoelectric properties in a solid solution of lead zirconate (PZ) and lead titanate (PT) at ∼45 mol% PT [7]. They attributed these enhanced properties to the MPB between rhombohedral and tetragonal PZT at that concentration (Figure 1).

Recent experimental work by Noheda *et al.* has suggested that a monoclinic phase exists at compositions very near the MPB [9], initiating a significant number of experimental and theoretical works to either support or refute this claim. To date, there is still controversy in the field as to the nature of the MPB in PZT. A few good reviews on this subject can be found in reference [10,11]. Nevertheless, the presence of an MPB is considered a necessary component of a lead-free replacement material because of the enhancement in properties observed in compositions near MPBs.

## 4. One Component Systems

Useful lead-free materials are often binary or ternary solid solutions. Before discussing these more complex material systems in Sections 5 and 6, this section introduces and discusses the respective end member compositions.

### 4.1. Barium Titanate—BaTiO_3_ (BT)

Barium titanate was one of the first useful piezoelectric materials, developed in the 1940s and 1950s [8,12,13]. Although this material does not have a very high piezoelectric constant, it has a very high permittivity, making it a good material for capacitors [14]. BT is often used in solid solution with other lead-free compounds to form an MPB which can enhance the piezoelectric and dielectric properties. BT exhibits a relatively low Curie temperature, however, and therefore has not seen many developments in recent years for applications in piezoelectric devices.

**Figure 2.**
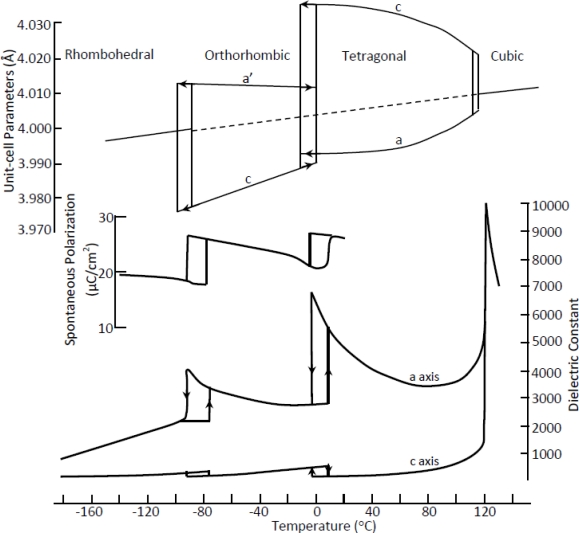
Polymorphic phase transitions in barium titanate single crystals observed through changes in the unit cell parameters [32], spontaneous polarization [33], and dielectric constant [15], reproduced from the respective sources.

The low Curie temperature of BT results from the tetragonal to paraelectric cubic phase transition at 120 °C [15]. BT undergoes several additional structural phase transitions upon decreasing temperature, as illustrated in Figure 2 (top). Each of these temperature-dependent phase transitions is referred to as a polymorphic phase transition (PPT), in contrast to the MPB which is composition-dependent. More recent studies by Wada *et al.* on single crystal BT found that these phase transitions can also be induced by applied electric field [16]. They found that the room temperature structure can change from tetragonal to monoclinic under an applied electric field amplitude of 10 kV/cm and then subsequently to rhombohedral at an amplitude of 30 kV/cm [16].

Recent developments in BT materials have reported extraordinary non-piezoelectric properties including piezoresistivity [17], colossal permittivity up to ∼10^6^ in carefully prepared nanocrystalline ceramics [18], and very large reversible strain up to ∼0.8% due to defect-mediated domain switching [19]. BT also continues to serve as a model system for more fundamental investigations, as in reference [20,21].

### 4.2. Sodium Bismuth Titanate—Na_0.5_Bi_0.5_TiO_3_ (NBT)

**Figure 3.**
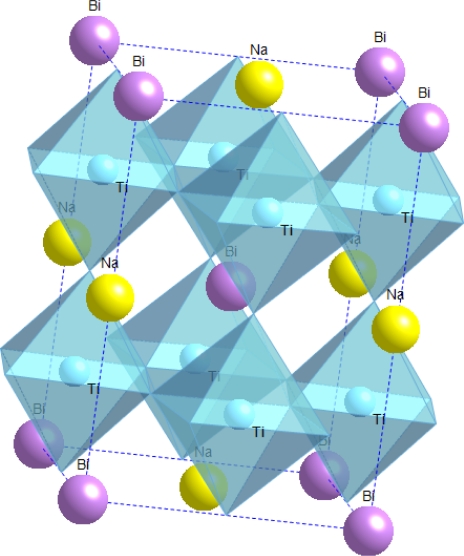
Schematic of the pseudo-cubic NBT perovskite structure. The structure illustrated is locally ordered on the A-site (Bi, Na), although the extent of ordering is not well known. The oxygen atoms are not shown for clarity and their positions are instead represented by the oxygen octahedra.

This material system, like several other lead-free materials, was first reported in the 1960s by Smolenskii *et al.* [22] but did not receive much attention until the recent surge in lead-free material development in the past two decades. Some of the initial dielectric and optical property measurements of NBT were reported in the 1990s by various sources [23–25]. Preliminary structural studies of NBT did not provide a definitive structural understanding [26,27], but in 2002 Jones and Thomas found that it expresses the rhombohedral *R3c* space group at room temperature and changes to tetragonal and subsequently cubic during heating [28]. NBT is a promising material due to its high Curie temperature of 325 °C, and a piezoelectric constant of 73 pC/N, similar to that of BT [29]. A schematic of the NBT structure above its Curie temperature is shown in Figure 3.

Some of the main drawbacks of this material are that it has a high coercive field and high conductivity. The issue of high conductivity was attributed to volatilization of Bi ions during sintering [29]. To address this issue, Hiruma *et al.* found an increased resistivity with the addition of excess bismuth [29]. NBT also exhibits a low depolarization temperature of 187 °C, far lower than its Curie point, limiting its use at elevated temperatures [29]. Some literature attributes this depolarization to the presence of an intermediate antiferroelectric phase [30,31], but other works claim that the intermediate phase is not fully antiferroelectric [29], leaving this question unresolved.

Different dopants can be added to NBT to combat some of its drawbacks, such as to decrease coercive field or increase the piezoelectric constant. When considering dopants for NBT, it is important to recognize that it differs from PZT in several ways. While PZT has complex ions on the B-site (Ti^4+^ and Zr^4+^), NBT has a mixture of Bi^3+^ and Na^+^ ions on the A-site. While PZT is therefore mainly B-site active, substitution in NBT is more effective on the A-site [34]. Many different studies have been done to test the impact of various dopants on NBT, some of which are tabulated in Table A.1 [35–38]. For example, an exceptional change was observed by Xiao *et al.* with the use of Li^+^ and K^+^ co-doping on the Na^+^ site [34,38]. Xiao *et al.* were able to increase the piezoelectric and coupling coefficients of NBT to 146 pC/N and 36%, respectively, and reduce the coercive field to 3.7 kV/mm while maintaining a high depolarization temperature to produce a lead-free middle frequency filter which performed comparable to a Pb-based one [34,38].

### 4.3. Potassium Bismuth Titanate—K_0.5_Bi_0.5_TiO_3_ (KBT)

**Figure 4.**
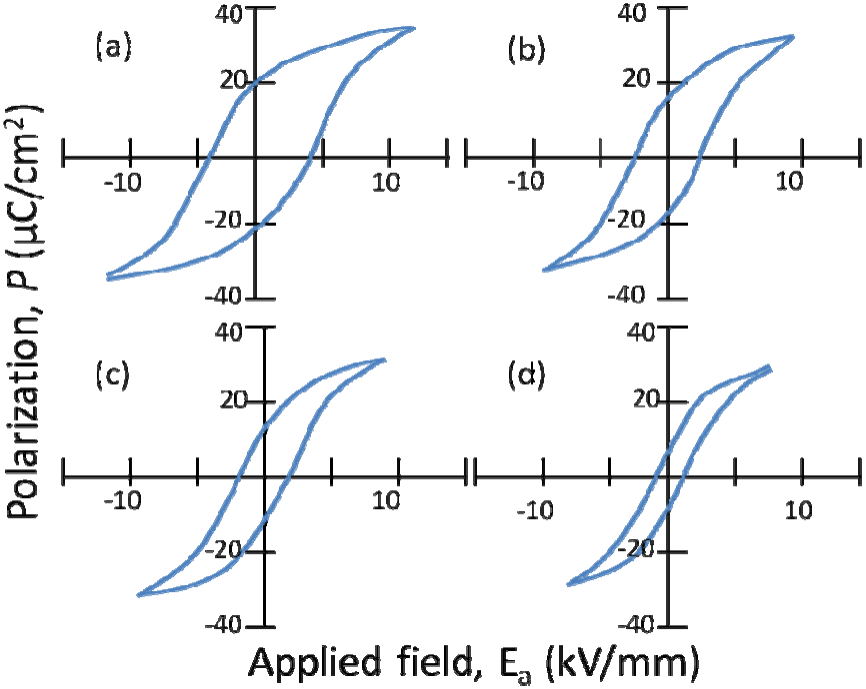
Polarization of KBT measured at temperatures of (a) 100 °C, (b) 200 °C, (c) 240 °C, and (d) 260 °C, reproduced from Hiruma *et al.* [39].

Similar to NBT, KBT was also first reported by Smolenskii *et al.* in the 1960s [22]. KBT is unique from NBT, however, in that it is tetragonal at room temperature (whereas NBT is rhombohedral) and does not depolarize until 270 °C [39] (NBT depolarizes at 187 °C). The polarization hysteresis behavior of KBT measured at several temperatures is displayed in Figure 4, showing that a hysteresis loop is observed even at temperatures as high as 260 °C [39]. As expected, the coercive field also gradually decreases with an increase in temperature, as does the remnant polarization. One of the main challenges of this system is that it is difficult to produce dense ceramics using ordinary firing methods. This low density makes the materials difficult to fully pole. Density can be improved through the use of sintering aids. For example, Hiruma *et al.* found that processing of KBT with excess bismuth oxide improved the piezoelectric and ferroelectric properties of the material (e.g., d_33_ = 101 pC/N) because Bi_2_O_3_ acts as a sintering aid and prevents the formation of micro-cracks [40].

### 4.4. Sodium Potassium Niobate—K_0.5_Na_0.5_NbO_3_ (KNN)

KNN is unique from the bismuth-based NBT and KBT compositions in that it is a specific composition (50/50) on a complete solid solution of NaNbO_3_ and KNbO_3_. This composition is at the MPB between two orthorhombic phases where KNbO_3_ is ferroelectric (FE) and NaNbO_3_ is antiferroelectric (AFE) [41,42], similar to the MPB observed in PZT. The structure of the material at the 50/50 composition is orthorhombic [43]. The piezoelectric coefficient of KNN is higher than that of undoped NBT or KBT [43], but not when processed through traditional processing methods [42]. Not only is the material difficult to sinter using ordinary conditions, but also the reactant powders require special care [44]. Dense ceramics with a high piezoelectric constant of 148 pC/N were achieved by Li *et al.* using advanced processing methods [43] compared to a piezoelectric constant of 90 pC/N for undoped KNN prepared using conventional methods [45].

**Figure 5.**
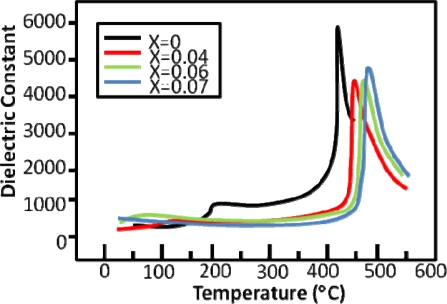
Dielectric constant of [Li_x_ (Na_0.5_K_0.5_)_1−x_]NbO_3_ measured as a function of temperature for several Li concentrations, reproduced from Guo *et al.* [45].

The processing issues of KNN did not make it an attractive contender for a PZT replacement, but the recent work of Saito *et al.* [3] promoted much research on this system. Saito *et al.* found that extremely high piezoelectric properties can be achieved relative to typical actuator-grade PZT compounds by combining the addition of several dopants with crystallographic texturing [3]. Following the publication of that paper, a great deal of literature on doping effects in KNN has been published. With the addition of dopants such as lithium, tantalum, and antimony, high density samples of KNN have been produced using traditional sintering. In some cases, the dopants are also used to improve the piezoelectric properties by decreasing the orthorhombic to tetragonal PPT to room temperature [44,46,47]. The aim of combining a material composition which contains the natural MPB at the 50:50 Na:K ratio with a PPT that is lowered to room temperature through the use of dopants is that the properties will be enhanced. Measurements of the dielectric constant with respect to temperature in Figure 5 show a peak at the PPT from orthorhombic to tetragonal, analogous to the behavior seen in BT in Figure 2. As certain dopants are added to decrease this temperature, the peak shifts down and eventually vanishes [45]. This is beneficial because an increase in the dielectric constant is correlated to an increase in the piezoelectric constant through the equation:

(5) $$d_{\mathrm{ij}}∼2Q_{\mathrm{ij}}\varepsilon_{r}\varepsilon_{o}P_{i}$$

where *d_ij_* is the piezoelectric coefficient, *Q_ij_* is the electrostriction coefficient, *ε_r_* is the relative permittivity, *ε_o_* is the permittivity of free space, and *P_i_* is the polarization [48].

### 4.5. Bismuth Ferrite—BiFeO_3_ (BFO)

**Figure 6.**
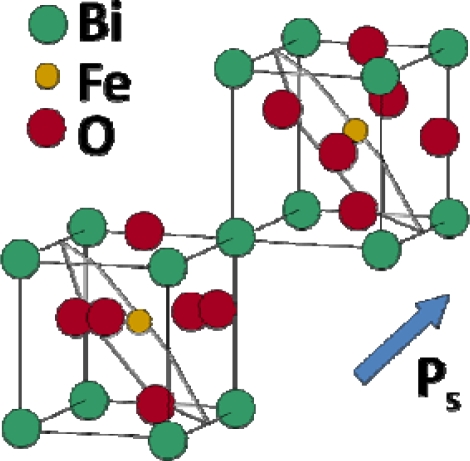
Schematic of the crystal structure of BFO, showing the direction of spontaneous polarization (P_s_) as well as the antiferromagnetic ordering (displayed in the grey plane), reproduced from Chu *et al.* [51].

Another intriguing system currently under study is bismuth ferrite. BFO is a unique lead-free candidate in that it is both ferroelectric and ferromagnetic. The structure, which has a bulk rhombohedral symmetry at room temperature, has spontaneous polarization mostly due to the bismuth on its A-site and magnetization due to the iron on the B-site [49]. Contrary to the bulk rhombohedral structure, BFO thin films have a monoclinic crystal structure [50]. A schematic of the structure, reproduced from Chu *et al.* in Figure 6, illustrates the spontaneous polarization direction as well as the planes of antiferromagnetic ordering [51]. Due to the co-existence of ferroelectricity and anti-ferromagnetism in this structure, a magnetic field can be used to change the orientation of the ferroelectric domains or, vice versa, an electric field can be used to change the ferromagnetic orientation [51].

In thin film studies, Wang *et al.* found that BFO has promising properties, with a remnant polarization of 50–60 μC/cm^2^ and a piezoelectric coefficient of 70 pm/V [50]. Fujino *et al.* attempted to further improve on these properties through doping with samarium and found an MPB at 14 mol% Sm between FE rhombohedral and AFE pseudo-orthorhombic structures [52]. This composition led to a decrease in the coercive field as well as an increase in the piezoelectric constant (110 pm/V) [52]. Developments in BFO have also extended to single crystals, where an extremely large spontaneous polarization of 100 μC/cm^2^ in the [001] direction was reported for highly pure single crystals [53]. Although recent work with BFO has shown great potential for its use, there are still many unanswered questions about its behavior, such as the lack in understanding of the BFO phase diagram and switching processes [49].

## 5. Binary Systems

For some of the lead-free systems described above, the piezoelectric properties are enhanced through the use of dopants. As mentioned previously, however, another useful method for the enhancement of properties is the selection of a composition near an MPB in a solid solution. This section discusses the structure and properties of several lead-free binary solid solution material compositions.

### 5.1. NBT-KBT

**Figure 7.**
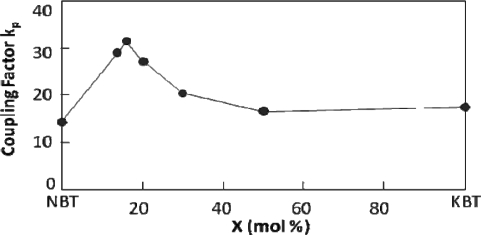
Electromechanical coupling factor of [Bi_0.5_(Na_1−x_K_x_)_0.5_] TiO_3_ as a function of KBT concentration (x), reproduced from Sasaki *et al.* [54].

A solid solution of NBT – KBT forms an MPB between rhombohedral (NBT-rich) and tetragonal (KBT-rich) structures in the region of 16–22 mol% KBT [54,55]. Compositions near the MPB show an improvement in certain properties such as the piezoelectric constant, dielectric constant, and the coupling factor, many of which are summarized in Table A.1 [54]. Figure 7 shows an example of the change seen in the coupling factor as a function of composition in the NBT-KBT solid solution.

Since this solid solution contains several elements that volatilize easily, such as K, Na, and Bi, the sintering conditions used play a large role in the final piezoelectric properties achieved in these materials [55]. For example, Zhang *et al.* found that with a change of only 40 °C in the sintering temperature of an NBT-KBT solid solution at the MPB, the piezoelectric constant increased from 155 to 192 pC/N [55].

### 5.2. NBT-KNN

In the case of a combination of NBT with KNN, several different MPBs have been reported. In an NBT-rich system, Kounga *et al.* reported an MPB at 6–7 mol% KNN between a rhombohedral FE phase (NBT-rich) and a tetragonal AFE phase [56]. Although the addition of KNN made the material more antiferroelectric in its behavior, it also led to a much higher unipolar strain [56]. On the contrary, with a KNN rich solution, an MPB was reported at 2–3% NBT between ferroelectric orthorhombic and tetragonal phases [57]. This composition leads to a high piezoelectric constant of 195 pC/N and electromechanical coupling factor of 43% [57].

### 5.3. NBT-BT

**Figure 8.**
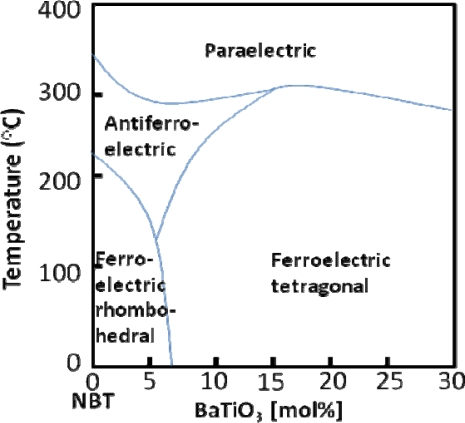
Phase diagram of NBT-BT showing the MPB between the ferroelectric rhomboheral phase and the ferroelectric tetragonal phase, reproduced from Takenaka *et al.* [58].

BT has also been combined in a solid solution with NBT, in which case an MPB is found at 6–7 mol% BT between the NBT-rich rhombohedral and BT-rich tetragonal phases [58]. At this composition, the system exhibits improved properties relative to NBT, such as the piezoelectric constant (d_33_ = 125 pC/N), coupling factor (k_p_ = 20%), and dielectric constant (*ε*_r_ = 580). NBT-BT compositions near the MPB also exhibit high bending strength (e.g., 200 MPa), which is 2–3 times that of PZT based materials [58]. One of the drawbacks of this system is the temperature dependence of the properties. As shown below in Figure 8, the MPB in NBT-BT is not linear with temperature, but rather has a curved shape [58]. It can be observed that the FE to AFE transition temperature decreases as the concentration of BT increases [58]. The behavior seen in the phase diagram was confirmed in more recent work by Hiruma *et al.* through electrical measurements, such as the temperature dependence of the dielectric constant and loss tangent, of various compositions of NBT-BT [59]. Daniels *et al.* found that compositions near the MPB exhibit electric field induced phase transitions from rhombohedral to tetragonal [60], similar to the electric-field-induced phase transitions mentioned earlier for BT. This behavior results in a strong orientation of the ferroelectric and ferroelastic domain orientations relative to the electric field direction in the material [60].

### 5.4. NBT-BFO

NBT was also combined with BFO by Nagata *et al.*, showing an increase in the Curie temperature (T_c_ = 340 °C) and the electromechanical coupling factor (k_p_ = 33.6%) [61] compared to undoped NBT (T_c_ = 325 °C, k_p_ = 16.8%) [29]. However, in this case, the structure remains a single rhombohedral phase with no MPB [61]. A change from the NBT system is observed at high temperatures of 200 °C, where the binary solution does not show the FE to AFE transition found in NBT [61].

### 5.5. KBT-BT

As mentioned previously, a major drawback of KBT is the difficulty in synthesizing dense ceramics using conventional processing methods. When KBT is in a solid solution with BT, the density of the material is improved [62]. With the addition of 10 mol% BT, dense textured materials were formed using ordinary sintering methods [62]. The properties for this system (e.g., d_33_ = 85 pC/N) [62] were comparable to those reported for KBT obtained through more advanced processing methods (d_33_ = 70 pC/N) [39].

### 5.6. KNN-BT

The addition of BT to KNN also aids in the densification of KNN-based materials, and Ahn *et al.* found improved piezoelectric properties for this system by comparing different sintering conditions of 0.95KNN-0.05BT [63]. Guo *et al.* reported an MPB in this system at ∼6 mol % BT and another transition to a cubic phase at 20 mol % BT [64]. KNN-BT systems were also prepared with Li doping, the results of which showed that higher piezoelectric coefficients were found at MPB compositions which were tetragonal-rich [65]. Table A.1 lists the properties of two KNN-BT systems, 0.95 KNN-0.05 BT and Li doped 0.90 KNN-0.10 BT, and both cases yield a piezoelectric constant above 200 pC/N [63,65].

## 6. Ternary Systems

As the binary systems described above still have many drawbacks and are not capable of replacing PZT in all of its applications, new lead-free compositions have become even more complex through the use of ternary solid solutions. Since the various end members described in Section 4 exhibit the perovskite structure, their combination is mostly expected to form solid solutions. Ternary systems also allow for more degrees of freedom in identifying compositions with enhanced properties.

**Figure 9.**
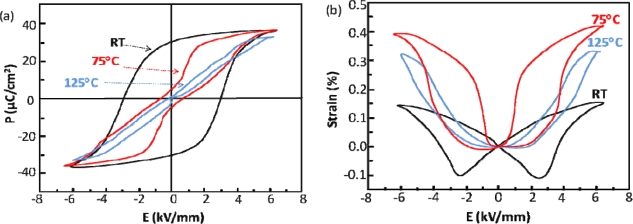
Polarization (a) and strain (b) loops of 0.93 NBT-0.05 BT-0.02 KNN ceramics at several temperatures, reproduced from Zhang *et al.* [67].

Several reports have been published by Zhang *et al.* for the NBT-BT-KNN ternary system [66,67]. The MPB in this system is between FE and AFE phases, where compositions such as 0.94 NBT-0.05 BT-0.01 KNN, 0.93 NBT-0.05 BT-0.02 KNN, 0.92 NBT-0.05 BT-0.03 KNN, and 0.93 NBT-0.06 BT-0.01 KNN show FE behavior, while 0.92 NBT-0.06 BT-0.02 KNN, 0.91 NBT-0.06 BT-0.03 KNN, 0.92 NBT-0.07 BT-0.01 KNN, 0.91 NBT-0.07 BT-0.02 KNN, and 0.90 NBT-0.07 BT-0.03 KNN show AFE behavior [66]. The ternary solid solution of 0.92 NBT-0.06 BT-0.02 KNN had the largest strain ever reported for a polycrystalline lead-free ceramic, ∼0.45% [66]. Further temperature dependent studies of the material found that this large strain comes from an electric-field-induced phase transition from FE to AFE rather than piezoelectric lattice distortion [67]. Temperature dependent strain and polarization loops of the MPB composition show that at higher temperatures the polarization loops begin to express AFE behavior as the strain loops display unusually large strain, shown in Figure 9 [67].

Studies of another ternary mixture, NBT-KBT-BT, at MPB compositions show that there is a trade-off between piezoelectric properties and depolarization temperature [68,69]. Generally, materials with higher piezoelectric properties exhibit lower depolarization temperatures. Wang *et al.* attempted to break this trend by finding the optimal amount of KBT to increase properties without the reduced depolarization temperature [70]. They found that the addition of ∼5 mol% KBT to a 0.95NBT-0.05BT mixture enhanced the piezoelectric coefficient (d_33_ = 148 pC/N) without having a strong impact on the depolarization temperature [70]. Recently a solid solution of NBT-KBT-KNN was examined by Yao *et al.* and an MPB was found for (Bi_1/2_Na_1/2_)_1−x_ (Bi_1/2_K_1/2_)_x_ TiO_3_ – 0.03 (Na_0.5_K_0.5_) NbO_3_ at x = 0.20 – 0.24 [71]. At a composition of x = 0.22, improved piezoelectric properties are found with a relatively high Curie temperature (d_33_ = 167 pC/N, T_c_ = 340 °C), showing a high potential for these compositions [71].

## 7. Summary

Over the past few years there have been many developments in the field of lead-free piezoelectric materials. Several material systems have been explored, some of which show properties comparable to PZT. However, there is still no single system that is as versatile in its applications as PZT. BT is historically significant and will continue to be a fundamental system of investigation, but it does not have properties comparable to PZT and is especially limited by its low Curie temperature of 120 °C. NBT has a higher Curie temperature, but lacks high enough piezoelectric properties and depolarizes below 200 °C. While KBT and KNN do not depolarize below their Curie points, they are difficult to process into dense ceramics using conventional sintering methods. The addition of dopants to KBT and KNN systems allows for easier processing of the materials. BFO is a unique material that has both ferroelectric and ferromagnetic properties. Recent developments show improvement in the piezoelectric properties for BFO thin films and single crystals.

Solid solutions containing MPBs such as KNN-BT and NBT-BT-KNN show promise, particularly when coupled with substitutional doping or positioning of the PPT temperature. Ultimately, more complex solid solutions and doping schemes will have to be explored because these provide an increasing number of degrees of freedom for identifying extraordinary properties. The materials reviewed in this paper form a foundation for the search for improved lead-free materials and show great potential for the future.

## Appendix A

**Table A. 1.** Summary some relevant properties of various lead-free materials.

Material	*d_33_* (pC/N)	*ε_33_^T^/ε_0_* (meas. freq)	*k_p_* (%)	*P_r_* (μC/cm^2^)	*E_c_* (kV/mm)	*T_c_* (°C)	Reference
BaTiO_3_ single crystal	85.6	168	-	-	-	-	[14]
BaTiO_3_ ceramic	191	1,680	-	-	-	-	[14]
BaTiO_3_ single crystal Parallel to [001] Parallel to [111] At high field -	125 203 295	-	-	-	-	-	[16]
Bi_0.5_Na_0.5_TiO_3_	72.9	343 (1 MHz)	16.8	-	-	325	[29]
1%Mn:NBT <001> oriented single crystal	130	-	-	-	-	-	[35]
[(Na_0.5_Bi_0.5_)_1−1.5x_V_0.5x_La_x_] TiO_3_ x = 0.01	88	-	15.4	-	-	-	[36]
[Na_0.5_Bi_0.5−x_La_x_] TiO_3_ x = 0.01	68	-	13.8	-	-	-	[36]
(Bi_0.5_Na_0.5_)_(1−1.5x)_La_x_TiO_3_ x = 0.0172	91	550 (1 kHz)	13	-	-	345	[37]
[Bi_0.5_(Na_1−x−y_K_x_Li_y_)_0.5_] TiO_3_ x = 0.15, y = 0.075	164–231	1,190	36.3–41.0	38.8–40.2	2.47–3.73	-	[38]
[Bi_0.5_(Na_1−x−y_K_x_Li_y_)_0.5_] TiO_3_ x = 0.15, y = 0.075	146	-	36	38.9	3.7	-	[34]
Bi_0.5_K_0.5_TiO_3_	69.8	517 (1 MHz)	-	22.2	5.25	437	[39]
KBT + 0.6wt% Bi_2_O_3_	101	764 (1 MHz)	-	27.6	5.30	391	[40]
Na_0.5_K_0.5_NbO_3_	148	559 (100 kHz)	38.9	-	-	395	[43]
Na_0.5_K_0.5_NbO_3_	70	400	25	-	-	-	[42]
(Na_0.5_K_0.5_)_1−x_ (LiSb)_x_Nb_1−x_O_3_ x = 0.052	286	1,372	51	-	-	385	[46]
[Li_x_(Na_0.5_K_0.5_)_1−x_] NbO_3_ x = 0.06	235	-	42	-	-	460	[45]
(Na_0.5−x/2_,K_0.5−x/2_,Li_x_)NbO_3_ (7%Li)	240	950 (1 kHz)	45	-	-	460	[44]
(Na_0.5−x/2_,K_0.5−x/2_,Li_x_) (Nb_1−y_Ta_y_) O_3_ x = 3,y = 20	190	920 (1 kHz)	46	-	-	310	[44]
(K_0.44_Na_0.52_Li_0.04_) (Nb_0.84_Ta_0.10_Sb_0.06_) O_3_	300	-	-	-	-	253	[3]
(K_0.44_Na_0.52_Li_0.04_) (Nb_0.84_Ta_0.10_Sb_0.06_) O_3_ textured	416	1,570 (1 kHz)	61	-	-	253	[3]
0.95 (Na_0.5_K_0.5_) NbO_3_-0.05LiTaO_3_	200	570 (10 kHz)	36	9	1.25	-	[47]
BiFeO_3_	15 – 60	∼30 (GHz)	-	-	-	-	[49]
BiFeO_3_ thin film	70	-	-	50 – 60	-	-	[50]
BiFeO_3_ single crystal	-	-	-	100	1.2	870	[53]
BiFeO_3_ ceramic	50 – 60	-	-	40	-	-	[72]
Bi_0.86_Sm_0.14_FeO_3_ thin film	110	-	-	70	-	-	[52]
[Bi_0.5_(Na_1−x_K_x_)_0.5_] TiO_3_ x = 0.16 x = 0.20	-	(100 kHz) 635 1,030	31.4 27.0	-	-	-	[54]
[Bi_0.5_(Na_1−x_K_x_)_0.5_] TiO_3_ x = 0.22	192	1,007	32.5	19.5	-	-	[55]
0.94Bi_0.5_Na_0.5_TiO_3_–0.06K_0.5_Na_0.5_NbO_3_	∼94	-	∼26	37	3.6	-	[56]
0.97(Na_0.5_K_0.5_) NbO_3_–0.03(Bi_0.5_Na_0.5_) TiO_3_	195	-	43	-	-	375	[57]
0.995(Bi_1/2_Na_1/2_) TiO_3_–0.005BiFeO_3_	-	530–700 (1 MHz)	-	33.6	6	340	[61]
(Bi_1/2_Na_1/2_)_0.94_Ba_0.06_TiO_3_	125	580 (10 kHz)	-	20	-	288	[58]
(0.9)(Bi_1/2_K_1/2_) TiO_3_–0.1BaTiO_3_ electric field applied parallel to tape stacking direction	84.5	560 (1 kHz)	-	-	-	-	[62]
0.90 (K_0.48_Na_0.48_Li_0.04_) NbO_3_–0.10BaTiO_3_	206	∼530	∼38	-	-	∼38 0	[65]
0.95 (Na_0.5_K_0.5_) NbO_3_–0.05BaTiO_3_	225	1,058	36	-	-	-	[63]
0.92NBT-0.06BT-0.02KNN	30 576*	2,320 (10 kHz)	-	16	1.3	-	[66]
0.93NBT-0.05BT-0.02KNN	98 276*	2,060 (10 kHz)	-	32	3.1	-	[66]
(0.90)(Bi_1/2_Na_1/2_)TiO_3_–0.05(Bi_1/2_K_1/2_) TiO_3_–0.05BaTiO_3_	148	700 (1 kHz)	34	35.9	-	-	[70]
0.852(Bi_1/2_Na_1/2_) TiO_3_–0.028BaTiO_3_–0.12(Bi_1/2_K_1/2_) TiO_3_	191	1,141 (1 kHz)	33	-	-	301	[69]
0.88NBT–0.08KBT–0.02BT (MPB)	181	-	-	-	-	300	[68]
0.78NBT–0.146KBT–0.074BT (tetragonal)	128	-	-	-	-	300	[68]
(Bi_1/2_Na_1/2_)_0.78_ (Bi_1/2_K_1/2_)_0.22_TiO_3_–0.03(Na_0.5_K_0.5_) NbO_3_	167	-	35.5	27.6	2.79	340	[71]

* Values are measured under large driving signals.
